# Symptomatic sinus bradycardia due to electrolyte imbalances in syndrome of inappropriate antidiuretic hormone (SIADH) related covid-19: a case report

**DOI:** 10.1186/s12879-021-06143-2

**Published:** 2021-05-21

**Authors:** Muzakkir Amir, Andi Renata, Levina Tri Ratana

**Affiliations:** 1 Departement of Cardiology and Vascular Medicine, Hasanuddin University, Makassar, Indonesia; 2 Dr. Wahidin Sudirohusodo National General Hospital, Makassar, Indonesia

**Keywords:** Symptomatic sinus bradycardia, Electrolyte imbalances, SIADH, COVID-19

## Abstract

**Background:**

Coronavirus Disease-2019 (COVID-19) has been declared a global pandemic since March 11th, 2020. Despite emerging reports and literature covering a broad spectrum of COVID-19 clinical manifestations, facets of COVID-19 have not been fully elucidated. To the authors’ concern, sinus bradycardia as a manifestation of COVID-19-induced syndrome of inappropriate antidiuretic hormone (SIADH) has never been reported before.

**Case presentation:**

In this paper, we report a case of a 59-year-old male patient with confirmed COVID-19 initially presented with presyncope. Further investigations reveal sinus bradycardia related to COVID-19-induced SIADH. This case highlights the possibility of immuno-neuroendocrino-cardiovascular crosstalk resulting in an atypical manifestation of COVID-19: near syncope due to sinus bradycardia.

**Conclusions:**

Another possible cause of sinus bradycardia in COVID-19 is electrolyte imbalance due to COVID-19-related SIADH.

## Background

COVID-19 is a terminology explaining a disease caused by Severe Acute Respiratory Syndrome-Coronavirus 2 (SARS-CoV2). COVID-19 has been declared a global pandemic since March 11th, 2020 [1]. By September 11th, 2020, COVID-19 has spread across 140 countries and has caused over 28.3 million confirmed cases and responsible for nearly 1 million deaths since its first occurrence in December 2019 [2]. COVID-19 were initially considered as primary lung disease, causing severe pneumonia and rapidly fatal acute respiratory distress syndrome (ARDS). Further investigations showed that COVID-19 manifests as several different phenotypes, involving some other organs besides the lungs themselves [3].

SIADH is frequently found in patients with pneumonia and other types of pulmonary infections. Distinguished by euvolemic hyponatremia, decreased plasma osmolality, increased urinary osmolality, elevated natriuresis in the absence of any different potential etiology, SIADH in patients with COVID-19 is currently recognized as a “clue in the times of pandemic” [4, 5]. Presence of SIADH should raise suspicion towards SARS-CoV2 infection. Hyponatremia resulting from COVID-19-induced SIADH has been reported to manifest as neurologic symptoms or as an asymptomatic one [6]. However, in our patient, it manifests primarily as symptomatic cardiac arrhythmia.

Electrolytes play a crucial role in the propagation of cardiac action potential [7]. Cardiac arrhythmias, one of which sinus bradycardia, have been noted as one of many phenotypes of COVID-19 and hypothesized to be caused by myocardial or pacemaker cell injury [8]. However, there was no previous report concerning symptomatic sinus bradycardia linked to hyponatremia in COVID-19 patients. Hyponatremia in COVID-19 patients is called “a possible immuno-neuroendocrine interface with COVID-19” [9]. Therefore, this case highlights the possibility of an immuno-neuroendocrino-cardiovascular interface with COVID-19 resulting in an atypical manifestation: near syncope due to sinus bradycardia.

## Case presentation

**Fig. 1:**
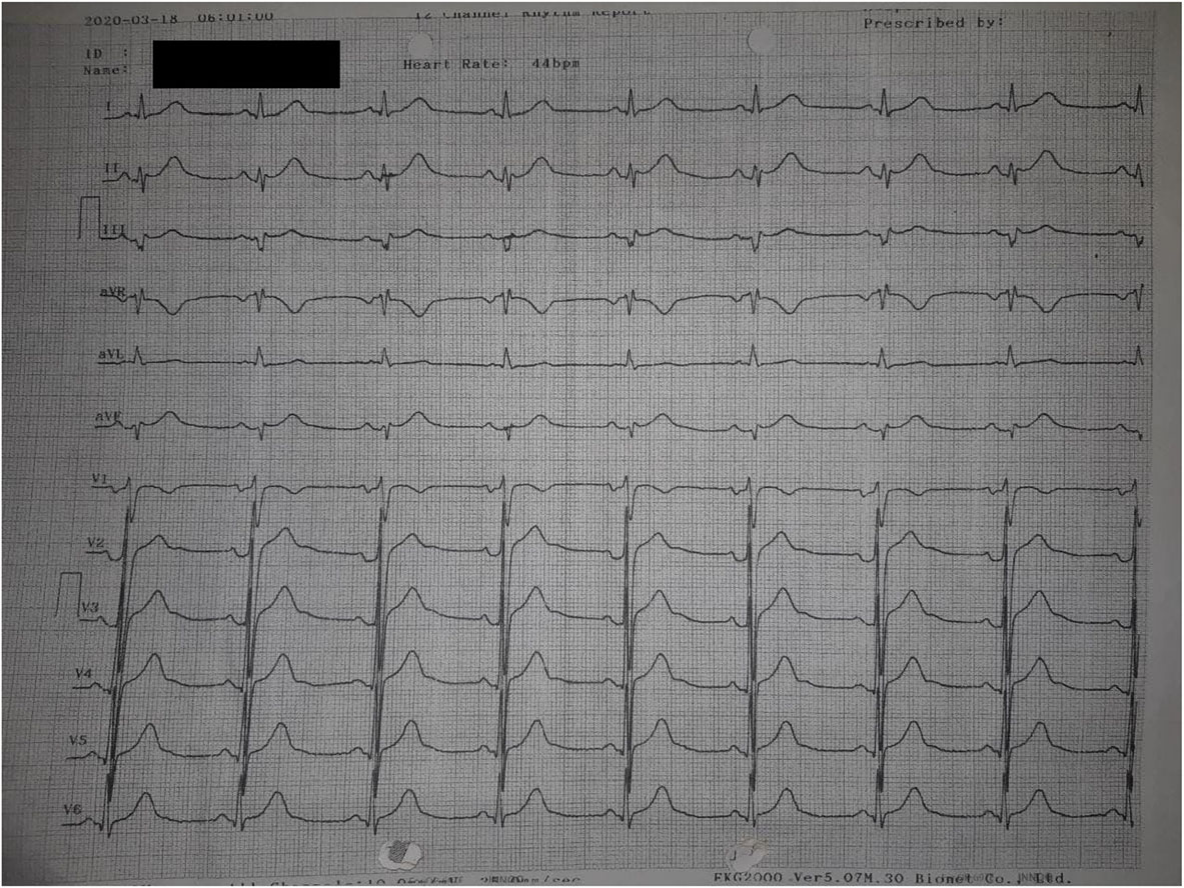
Electrocardiography showing sinus bradycardia and 44 bpm heart rate

A 59-year-old male presented to the emergency unit of dr. Wahidin Sudirohusodo General Hospital with complaints of recurrent near-syncope, which occurred a couple of hours ago. He described the episode as light-headedness followed by a brief blackout without loss of consciousness. He also had complaints of weakness and fatigue. He had no previous history of similar episodes. He denied any respiratory problems, fever, or close contact with COVID-19 confirmed patients. To his knowledge, he has no underlying cardiovascular conditions and was otherwise healthy. During the examination, he was afebrile, with a blood pressure of 144/88 mmHg, a heart rate of 44 beats per minute, and a respiratory rate of 20 breaths per minute with oxygen saturation of 94% on room air. Physical examination was nothing out of the ordinary, with no signs of volume overload or dehydration. Electrocardiogram (ECG) on admission (Fig. 1) showed sinus bradycardia with 44 beats per minute. High sensitivity Troponin I assay showed 3.8 ng/L. A 2-h repeated assay was performed with a result of 4.9 ng/L.

Laboratory findings were significant for serum sodium of 117 mmol/L (reference range 136–145 mmol/L), serum potassium of 3.4 mmol/L (reference range 3.5–5.0 mmol/L) and increased neutrophil/lymphocyte ratio 8,36 (78.6/9.4). Blood urea nitrogen was 11.7 mg/dL (reference range: 7–20 mg/dL) and random blood glucose was 122 mg/dL, resulting in calculated plasma osmolality of 245 mOsm/kg (reference range: 275–299 mOsm/kg). Further workup revealed urine osmolality of 142 mOsm/kg (reference range: 500–850 mOsm/kg) and urinary sodium 34 mmol/L with low plasma osmolality. Thyroid-stimulating hormone and Free T4 were normal (1.77 mIU/mL and 1.41 ng/dL consecutively). The patient was planned to be admitted to the Cardiology Care Unit. However, during the routine COVID-19 screening examination, chest CT scan revealed multiple ground-glass opacities typical for COVID-19 pneumonia. Following the chest CT result, a naso- and oropharyngeal swab for SARS-CoV-2 PCR was obtained from the patient, which came back positive with an IL-6 result of 13.2 pg/ml. Further workup fulfilled the criteria for SIADH, and other potential causes of hyponatremia were excluded. No other potential etiology of SIADH was found, and the patient was assessed to have a COVID-19-associated SIADH.

The patient was treated with a slow infusion of hypertonic saline and oral potassium supplementation. After the serum sodium concentration normalized, his symptoms and heart rate improved (Table 1), and further ECG monitoring showed no bradycardic events. The patient was given azithromycin and oseltamivir from the pulmonology department. Despite his asymptomatic state, the nasopharyngeal swab test remains positive until the 30th day of hospitalization. This case is the first case report concerning symptomatic sinus bradycardia related to COVID-19 associated SIADH to the author’s concern.

**Table 1:** Course of Sodium/Potassium and Electrographic changes

Hospitalization day	Na^+^ (mmol/L)	K^+^ (mmol/L)	ECG Interpretation
*1st*	*117*	*3.4*	*Sinus Bradycardia, 40 bpm*
*3rd*	*118*	*3.7*	*Sinus Bradycardia, 50 bpm*
*5th*	*132*	*4.1*	*Normal Sinus Rhythm, 60 bpm*
*8th*	*136*	*4.2*	*Normal Sinus Rhythm, 75 bpm*
*11th*	*134*	*4.2*	*Normal Sinus Rhythm, 72 bpm*
*18th*	*135*	*4.0*	*Normal Sinus Rhythm, 60 bpm*

## Discussion

Bradyarrhythmias could be an early manifestation of various cardiac and extracardiac conditions. COVID-19 has been linked to sinus bradycardia by several past reports. Amaratunga et al. reported a group of 4 patients experiencing sinus bradycardia during hospitalization for COVID-19. Extreme hypoxia, inflammatory damage to cardiac pacemaker cells, and an exaggerated reaction to medication were all listed as contributing factors to bradycardia in this study [8]. In contrast to the abovementioned mechanism, bradycardia in our patient occurs under normal oxygen saturation at room air, and the patient was not under any medication. Direct myocardial injury was also excluded from normal 0-h and 2-h high-sensitivity troponin I assay. Significant laboratory result was only electrolyte imbalance. Inflammatory cytokines could also exert a direct effect on pacemaker cells. However, our patient’s inflammatory marker did not increase during the bradycardic episode. Furthermore, the patient did not develop any inflammatory or respiratory symptoms until the end of hospitalization. Therefore, symptomatic sinus bradycardia in our patient may be related to other pathways rather than the abovementioned mechanism.

Cardiac arrhythmias related to hyponatremia usually occur in the setting of underlying heart disease, antiarrhythmic drugs, or concomitant with other electrolyte abnormalities. Although the exact mechanism is not clear, severe hyponatremia has been associated with cardiac conduction defects through the genesis action potential. Karabag et al. reported a case of severe hyponatremia with complete heart block that is fully reversible upon normalization of sodium levels, and previously Mouallem et al. also have reported a case series including hyponatremia and various cardiac arrhythmia manifestations [10, 11].

Low extracellular sodium could reduce inward sodium flow in the early phase of the cardiac action potential. Trautwein et al. showed that low sodium levels in isolated heart muscle perfusate have a negative chronotropic and dromotropic effect [12]. A study by Nikolaidou et al. showed that low sodium levels could affect action potentials in the atrioventricular node [13]. Association between hyponatremia and dysautonomia has also been reported in various other conditions such as Guillain-Barre syndrome and porphyrias [14, 15]. Our patient has an associated electrolyte abnormality, which is mild hypokalemia. Yet, sinus bradycardia persists upon normalization of plasma potassium levels and improved upon hyponatremia correction.

Interleukin-6 (IL-6) plays an essential role in causing electrolyte imbalance by inducing the non-osmotic release of vasopressin [16]. Released by monocytes and macrophages, IL-6 will result in an inflammatory state which causes multiple organ failure. Berni et al. performed a preliminary study to confirm this condition, and they reported that IL-6 was inversely correlated with sodium levels. Hyponatremia was associated with more severe conditions (OR = 14.8, 95% CI 1.5 ± 144,2, *p* = 0.02) [17]. Although this study is still limited, we agreed that sodium levels must be considered an alternative biomarker in the clinical protocols for COVID-19 patient management and prognosis.

SIADH is characterized by euvolemic hyponatremia, low plasma osmolality, high urinary osmolality, elevated natriuresis, and lack of evidence of other hyponatremic diseases [4]. SIADH was frequently found in patients with pneumonia. A case series by Yousaf et al. highlights SIADH as a clue in the time of the pandemic [5]. Habib et al. and Yousaf et al. reported COVID-19 patients who develop SIADH without significant respiratory problems [5, 6]. COVID-19-induced stress stimulates the hypothalamo-hypophyseal axis, or hypothalamic neuron directly, and stimulates ADH release. In animal models, decreased osmolality cause osmoreceptor to fire and increase the ADH. Inflammatory cytokines could directly stimulate the non-osmotic release of ADH [5]. Another pathway in which an inappropriate amount of ADH could be secreted is via the hypoxic pulmonary vasoconstriction pathway [5], which might be unsuitable in our patient because the patient has no respiratory symptoms and have normal oxygen saturation. SIADH could result in dysautonomia [14].

## Conclusions

In this case, we present an atypical manifestation of COVID-19 in an otherwise healthy patient: near-syncope. It is important to note that bradycardia (and its related symptoms) related to electrolyte imbalance could be the initial and the only manifestation of COVID-19. Therefore, these findings should warrant the clinician to address this unusual presentation carefully to improve patient outcomes and prevent in-hospital COVID-19 transmission.

## Data Availability

The data that support the findings of this case report was taken from the dr. Wahidin Sudirohusodo Hospital’s medical record and are available from the corresponding author upon reasonable request.

## Abbreviations

COVID-19: Corona Virus Disease 19
SARS-CoV-2: Severe Acute Respiratory Syndrome-Coronavirus 2
RDS: Acute Respiratory Distress Syndrome
SIADH: Syndrome of Inappropriate Antidiuretic Hormone
CT scan: Computed Tomography Scan
IL-6: Interleukin-6
ECG: Electrocardiogram
0-h: 0-h; 2-h: 2-h
